# Mechanical history shapes phenotypic plasticity and fibroblast-autonomous 3D remodeling in iPSC-derived cardiac fibroblasts

**DOI:** 10.64898/2026.10.20.759862

**Published:** 2026-10-25

**Authors:** Huanzhu Jiang, Ganesh Malayath, Austin Kellogg, Nongmaithem Debeni Devi, Charlotte Chen, Nathaniel Huebsch

## Abstract

Human induced pluripotent stem cell-derived cardiac fibroblasts (iPSC-cFBs) offer a renewable, genetically defined stromal cell source for studying the biology of cardiac fibrosis and of cardiomyocyte-fibroblast crosstalk in the context of development and disease. As cardiac fibroblasts are proliferative cells, the ability to expand iPSC-cFB in culture is attractive given the need for large cell numbers for experiments such as drug screening and tissue engineering efforts. However, potential phenotypic drift during expansion – in particular, the prospect of a premature Fibroblast-to-Myofibroblast-Transition (FMT) is a concern. Here, we investigated how differentiation protocol, mechanical culture history, and serial expansion shape iPSC-cFB phenotype, transforming growth factor-β1 (TGF-β1) responsiveness, and fibroblast-autonomous remodeling in three-dimensional (3D) tissues. Initial studies of iPSC-cFBs expanded on conventional tissue-culture polystyrene (TCP) revealed passage-associated increases in projected cell area and expression of α-smooth muscle actin (α-SMA) two markers of FMT. These findings motivated us to study how the rigidity of a poly (dimethyl siloxane) (PDMS; either 15 kPa or 2 MPa) substrate used during initial iPSC-cFB derivation and subsequent expansion impacts both basal phenotype and response to the FMT-inducing growth factor TGF-β. Across iPSC-cFB derived with two independent differentiation protocols, mechanical conditioning produced passage-dependent basal phenotypes and responsiveness to TGF-β. Interestingly, the impact of mechanical pre-conditioning on collective response of iPSC-cFB to TGF-β within “ *in vivo* -like” 3D cultures was distinct from the impact of this conditioning when the cells were plated to 2D substrates. Together, these findings demonstrate that mechanical culture history shapes iPSC-cFB response capacity and tissue-remodeling behavior without irreversibly fixing fibroblast state. Passage and subsequent microenvironment can attenuate or re-express these history-dependent phenotypes. This study establishes a foundation for using iPSC-cFB for efforts in basic biological discovery and future tissue engineering efforts.

